# Meat, Meat Products and Seafood as Sources of Energy and Nutrients in the Average Polish Diet

**DOI:** 10.3390/nu10101412

**Published:** 2018-10-02

**Authors:** Wacław Laskowski, Hanna Górska-Warsewicz, Olena Kulykovets

**Affiliations:** Department of Organization and Consumption Economics, Faculty of Human Nutrition and Consumer Sciences, Warsaw University of Life Sciences, 02-787 Warsaw, Poland; waclaw_laskowski@sggw.pl (W.L.); olena_kulykovets@sggw.pl (O.K.)

**Keywords:** meats and seafood, energy intake, nutrient intake, food sources

## Abstract

The aim of this study was to identify the share of meat, meat products and seafood in the contribution of energy and 22 nutrients to the average Polish diet. Data from the nationally representative sample of Polish population (2016 Household Budget Survey) on meat and seafood product consumption from 38,886 households (*n* = 99,230) were calculated into one person per month. The analyses were conducted for seven food groups (e.g., red meat, poultry) and 16 products (e.g., beef, chicken). Approximately 18.5% of energy is delivered from the sources such as meat, meat products and seafood, providing a higher percentage of 18 nutrients to the diet (e.g., 56.0% of vitamin B12, 52.3% of niacin, 44.9% of cholesterol, 41.5% of protein, 41.4%of vitamin D, 37.6% of monounsaturated fatty acids (MUFA), 37.4% of thiamin, 33.8% of zinc, 32.0% of total fats, 30.3% of saturated fatty acids (SFA), 29.6% of vitamin B6, 25.3% of riboflavin, 24.9% of phosphorus, 24.8% of iron, 22.5% of vitamin A, 21.6% of polyunsaturated fatty acids (PUFA) and 20.3% of sodium). For the contribution of 18 nutrients and energy, processed meat products were ranked first. These results should be taken into consideration in order to compose diets with adequate energy and nutrient contribution and also to analyze benefits and risk resulting from the current level of consumption of red and processed meat, fish and other seafood.

## 1. Introduction

Currently, the role of meat in human nutrition is widely discussed in the scientific literature [1,2,3,4,5,6,7,8,9,10]. From one point of view, being a valuable source of macro- and micro-nutrients, particularly of bioavailable iron and zinc [10,11,12,13], vitamins B1, B12, niacin [10,12,13], proteins and vitamins A, D [10,13], meat has a very high nutritional value. It is underlined that heme iron is more efficiently absorbed from meat products (20–30%) than non-heme iron (5–15%) [13]. Meat contains high biological value protein [10,14] with all essential amino acids [13], better protein and amino acid digestibility and higher protein to energy ratios [15]. From the other point of view some epidemiologic and large-scale studies analyzed the association between consumption of red and processed meat and the occurrence of common disease, that is, type 2 diabetes [4,10,16,17], certain types of cancer [10], including esophageal, [18], ovarian [19], breast [20,21], esophagus and liver cancer [22]. Some findings underlined the long-term increasing consumption of red and processed meat leading to the increased risk of total mortality [10,23,24]. On the contrary, poultry consumption is associated with a risk reduction of developing overweight and obesity, cardiovascular diseases and type 2 diabetes. Poultry meat is considered moderately protective or neutral on cancer risk [25].

At the same time, total meat consumption in the U.S., European Union and other developed countries has increased [2,26]. As a very important part of everyday diet [27], meat contributes more than 15% to daily energy intake, 40% to daily protein intake and 20% to daily fat intake [1]. In the structure of meat consumption, pork and poultry together account for 80% of the total meat consumption in the European countries and their share increases [28]. In Poland, similar trends are observed. In 2000–2016, the increase of poultry consumption, the decrease of beef consumption and a high-level pork consumption were observed [29,30,31,32,33]. Due to the above mentioned conditions, the level and structure of meat consumption is the subject of many studies [1,2,3,26,34,35,36,37,38,39,40,41,42,43,44,45] leading to the identification of meat consumption patterns [46,47], health-related aspects [4,10,16,17,20,21,22,24,47,48,49,50,51,52,53] and factors determining the consumer’s choice including psychological [30,54,55,56,57,58], economic [30,58], social, health, education [30], macroeconomic (e.g., tax regulation influencing price level) and market conditions [27,59,60].

In many countries, including Poland, the increasing consumption of meat and meat products is accompanied by an increase in fish and seafood consumption [61,62,63,64,65,66]. This trend is determined by economic [67], psychological [61], religious [62], health [65,66,68,69] factors and product availability [67]. However, the increase in fish consumption leading probably to a decreased incidence of cardiovascular disease (due to long-chain n-3-fatty acids and vitamin D contribution) [69,70,71,72,73,74] should be considered with the increased concentrations of environmental toxins (polychlorinated biphenyls, poly and perfluoroalkyl substances and methylmercury) [73,74]. Contaminants detected in fish are associated with cardiovascular diseases, metabolic disorders and cancer [74].

Based on the above-mentioned arguments and conditions, it is pointed that the analysis of meats and seafood as sources of energy and nutrients is important to ensure the proper structure of the balanced diet and to increase the nutrient adequacy. The aim of this study was to identify the share of meat, meat products and seafood in the contribution of energy and 22 nutrients to the average Polish diet. This analysis was conducted using data from a nationally representative sample of the Polish population selected by the Central Statistical Office within 2016 Household Budget Survey.

## 2. Materials and Methods

### 2.1. Study Overview

Energy and nutrients intake from meat, meat products and seafood were analyzed. The 22 nutrients examined in this study included: protein, total fat, saturated fatty acids (SFA), monounsaturated fatty acids (MUFA), polyunsaturated fatty acids (PUFA), cholesterol, iron, zinc, potassium, sodium, phosphorus, calcium, copper, magnesium, vitamin A, vitamin D, thiamin, riboflavin, niacin, vitamin B6, vitamin B12 and vitamin E.

### 2.2. Sample Selection Method

Household Budget Survey (HBS) is based on the representative method conducted systematically by statistical offices. The survey is organized by the Central Statistical Office, Social Surveys and Living Conditions Statistics Department in cooperation with Statistic Office in Łódź which specializes in living condition statistic. In the survey, each participating household keeps records of expenditures, quantitative consumption and revenues in special budget books for one month [75,76].

**Figure 1:**
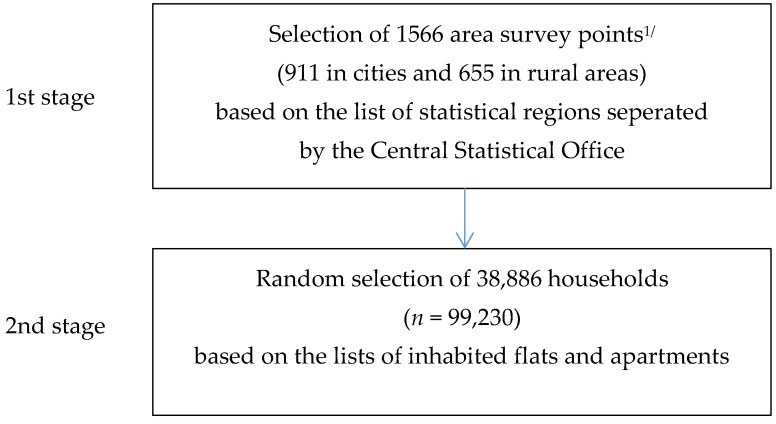
Study sample selection of 2016 HBS. ^1/^ area survey point = research area separated by the Central Statistical Office for purpose of the HBS and included at least 250 flats or apartments in the cities and 150 in the countryside. Source: based on the data of the Central Statistical Offices [75,76].

In order to collect a study sample, a two-stage layered scheme was used. The sampling units of the first stage were area survey points and in the second stage, flats and apartments were drawn. The basis for the sampling frame for the first-degree units was the list of statistical regions developed for the needs of the National Census, updated each year with changes resulting from the administrative division of the country. For each region included in the survey the information on the address features and the estimated data on the number of inhabitants and the number of flats were recorded. It was assumed that the area survey point in the city included at least 250 apartments, while in the countryside, it included at least 150 households. In 2016, 30,000 area survey points were created for the entire country. As a result, 1566 area survey points were obtained: 911 area survey points were located in cities and 655 in the rural areas (Figure 1). The second-degree sampling was made up of lists of inhabited flats and apartments in randomly selected area survey points, developed by statistical offices. The sources of data on each household participating in the survey were based on the “Budget Diary” and “Household’s Statistical Form”. The HBS was conducted by interviewers who were employees of statistical offices in voivodships [75,76]. In the 2016 HBS, 38,886 households (*n* = 99,230) participated in the survey [75].

Using the information on the number of persons in a household and the number of days of using home nutrition, data on the consumption of food products were converted for one person per month. Such converted data ingestion should be regarded as the comprehensive diet [77].

### 2.3. Food Grouping

The HBS included 91 food groups. There were (Table 1):

- 4 food groups (meat, poultry, other meat and meat products) and 12 detailed food products (e.g., beef, veal) in the meat and meat products category,
- 3 food groups (i.e., fishes, shellfish and processed seafood) and 4 detailed food products (i.e., dried, chilled and frozen fish; dried, chilled and frozen shellfish; dried, smoked and salted seafood; and other fish and shellfish products) in seafood category.

The food classification scheme was adapted from the studies published earlier [5,78,79,80]. For the purpose of this study, the food classification was modified to include the commonly consumed foods among the Polish consumers [75].

### 2.4. Statistical Analysis and Results Presentation

To calculate the energy and nutrient content of the food, the ”Nutritive Value Tables for Foods and Meals” [81] were used; tables from the fourth edition were developed and updated by the Food and Nutrition Institute located in Warsaw. From the base of 1100 products and assortment items, 930 products were selected. The average energy and nutrient content were calculated considering, if necessary, the weights resulting from the known or estimated proportion of the consumption of the product relative to the others in the group.

Using the R program (v 3.0.2), a system and an environment for the statistical computation [82,83,84], particularly, the action commands on arrays, matrices and vectors, the energy value and the nutrient content were calculated for the consumption of each of the 38,886 households (*n* = 99,230). Statistical calculations were performed with the weight of corrections to improve the representativeness of the results and the size of the household. This allowed us to recognize the results as representative for the population of Poland [77,85].

For the purpose of this study, the mean and the standard errors of energy were calculated for 7 food groups and 16 food products from the meats and the seafood categories. The mean nutrient intake was expressed as a percentage of the total dietary intake of the analyzed nutrient and presented in a ranked order. A two-stage method of presenting the results was adopted:

(1) the most important data related to the share of main food groups in contribution of energy and 22 nutrients intake presented in Section 3 “Results” in following order:
  Section 3.1—energy,
  Section 3.2—protein, total fats, fatty acids (SFA, MUFA, PUFA) and cholesterol,
  Section 3.3—micronutrients (iron, zinc, sodium, phosphorus, calcium, copper and magnesium),
  Section 3.4—vitamins (vitamin A, vitamin D, thiamin, riboflavin, niacin, vitamin B6, vitamin B12 and vitamin E).
(2) the detailed data related to the share of 7 food products and 16 food products in contribution of energy and 22 nutrients intake presented in Supplement Tables.

## 3. Results

The food sources of energy and 22 nutrients from two food categories (1—meat and meat products and 2—seafood) are shown in Table 2, Table 3, Table 4, Table 5, Table 6, Table 7, Table 8, Table 9 and Table 10 and Supplemental Tables S1–S23.

### 3.1. Meat, Meat Products and Seafood as Sources of Energy

The energy contribution of food groups from the meat and meat products and the seafood categories is presented in Table 2 and Table S1. The main food group contributors of energy from meat, meat products and seafood in the average Polish diet were meat products (8.3%), red meat (4.9%) and poultry (3.6%) (Table 2). When considering food products, the top three ranking foods were processed red meat products (6.8%), pork (4.7%) and chicken (3.2%) (Table S1).

### 3.2. Meat, Meat Products and Seafood as Sources of Protein, Total Fats, Fatty Acids and Cholesterol

The share of meat, meat products and seafood in the contribution of protein, total fat, SFA, MUFA, PUFA and cholesterol in the average Polish diet is presented in Table 3. The main food sources of these nutrients are shown in Table 4, while the detailed data of the food groups and food products are included in Supplemental Tables S2–S7.

Meats and seafood were the sources for approximately 41.5% of the protein in the average Polish diet (Table 3). The three highest sources of protein from the meats and the seafood categories were meat products (17.4%), red meat (9.9%) and poultry (9.7%) (Table 4). The highest ranked food products sources of protein were processed red meat products (14.0%), pork (9.2%), chicken (8.5%), other meat products (2.0%) and liver and organ meat (1.5%) (Table S2).

The main food groups as contributors to protein were also the top sources of total fat: meat products (14.7%), red meat (8.9%) and poultry (5.9%) (Table 4). When considering food products from meats and seafood, the main sources of total fat were processed red meat products (12.5%), pork (8.7%), chicken (5.2%), other meat products (1.5%) and liver and organ meat (1.1%) (Table S3).

Meats and seafood contributed 30.3% of SFA, 37.6% of MUFA and 21.6% of PUFA in the average Polish diet (Table 3). When considering the three main food groups, meat products, red meat and poultry contributed nearly 28.5% of SFA, 35.0% of MUFA and 18.7% of PUFA in the Polish diet (Table 4). The detailed data shows the highest ranked food sources of SFA, MUFA and PUFA, as presented in Tables S4–S6.

The cholesterol contribution from the meats and the seafood categories amounted to 44.9% (Table 3). The main food group contributors of cholesterol from meat, meat products and seafood in the average Polish diet were meat products (16.3%), poultry (11.8%) and red meat (8.3%) (Table 4). When considering food products, the top five ranking food were: processed meat products (12.3%), chicken (10.4%), pork (7.7%), liver and organ meat (5.7%) and other meat products (2.4%) (Table S7).

### 3.3. Meat, Meat Products and Seafood as Sources of Micronutrients

The shares of meat, meat products and seafood in the contribution of iron, zinc, sodium, phosphorus, calcium, copper and magnesium to the average Polish diet is presented in Table 5. The food sources of these micronutrients calculated in the main food groups are shown in Table 6 and the detailed data related to food groups and products are included in Supplemental Tables S8–S15.

Meats and seafood were the sources of nearly 25% of the iron in the average Polish diet (Table 5). The three highest sources of iron from the meats and the seafood categories were meat products (10.4%), red meat (4.7%) and poultry (4.3%) (Table 6). When considering food products from meats and seafood, the main sources of iron were processed meat products (7.7%), liver and organ meat (4.0%) and pork (4.0%) (Table S8).

Meats and seafood are a very important source of zinc, delivering 33.8% of the total intake (Table 5). The main food groups as the contributors to zinc were meat products (15.5%), red meat (9.4%) and poultry (5.6%) (Table 6). The highest ranked food product sources of zinc were processed meat products (12.9%), pork (8.5%), chicken (4.5%), liver and organ meat (1.9%), other meat products (1.7%) and poultry excluding chicken (1.1%) (Table S9).

Meats and seafood contributed 20.3% of the sodium (Table 5) in the average Polish diet. The top contributors of sodium from the main food groups were meat products (15.2%) and processed seafood (2.7%) (Table 6) and from the detailed food products, they were processed meat products (12.4%); dried, smoked and salted fishes and shellfish (2.0%); processed poultry products (1.4%); and other meat products (1.4%) (Table S10).

Meats and seafood were the sources for nearly 20% of the potassium in the average Polish diet (Table 5). The highest sources of potassium from the meats and the seafood categories were meat products (6.7%), red meat (5.2%) and poultry (5.0%) (Table 6). When considering food products from meats and seafood categories, the main sources of potassium were processed meat products (4.9%), pork (4.8%) and chicken (4.3%) (Table S11).

The phosphorus contribution from the meats and the seafood categories was 24.9% (Table 5). The main food group contributors of phosphorus from meat, meat products and seafood in the average Polish diet were meat products (9.0%), poultry (6.9%) and red meat (5.4%) (Table 6). When considering food products, the top five ranking foods were processed meat products (6.6%), chicken (5.9%), pork (5.0%), other meat products (1.2%) and processed poultry products (1.2%) (Table S12).

Meats and seafood were the sources of nearly 5% of the calcium in the average Polish diet (Table 5). Calcium was delivered mainly by meat products (Table 6). The detailed data of the food sources of calcium are presented in Table S13.

Meats and seafood contributed 13.7% of the copper (Table 5) in the average Polish diet. The three main food groups of meat products, other meat and poultry contributed nearly 10.2% of the copper (Table 6). The detailed data showed that the highest ranked food sources of copper were processed meat products (3.3%), liver and organ meat (3.0%), pork (2.0%) and chicken (2.0%) (Table S14).

Meats and seafood contributed 13.1% of the magnesium (Table 5) in the average Polish diet. The top contributors to magnesium were the following food groups: meat products (4.9%), poultry (3.7%) and red meat (2.8%) (Table 6), those from the detailed food products were processed meat products (3.3%), chicken (3.1%) and pork (2.5%) (Table S15).

### 3.4. Meat, Meat Products and Seafood as Sources of Vitamins

The shares of meat, meat products and seafood the contribution of vitamin A, vitamin D, thiamin, riboflavin, niacin, vitamin B6, vitamin B12 and vitamin E are presented in Table 7. The food sources of these vitamins from meat, meat products and seafood calculated in the food groups are shown in Table 8, while the detailed data related to the food groups and food products are included in Supplemental Tables S16–S23.

Meats and seafood contributed 22.5% of the vitamin A (Table 7) in the average Polish diet. The top contributors to vitamin A were the following food groups: other meat (17.3%) and meat products (3.3%) (Table 8). While considering the detailed food products, the highest sources of vitamin A were liver and organ meat (17.3%) and other meat products (3.0%) (Table S16).

Meats and seafood are a very important source of vitamin D, delivering 41.4% of the total intake (Table 7). The main food groups as the contributors to vitamin D were processed seafood (11.0%), meat products (8.8%) and poultry (8.7%) (Table 8). The highest ranked food product sources of vitamin D were chicken (8.0%), processed meat products (7.4%), pork (6.5%), dried, smoked and salted seafood (5.8%), other fish and shellfish products (5.2%) and fresh, chilled or frozen fish (5.1%) (Table S17).

Meats and seafood were the sources of nearly 37.4% of the thiamin in the average Polish diet (Table 7). The three highest sources of thiamin from the meats and the seafood categories were meat products (16.5%), red meat (16.0%) and poultry (2.7%) (Table 8). When considering food products from the meats and the seafood categories, the main sources of thiamin were pork (15.8%), processed meat products (14.7%) and chicken (2.4%), (Table S18).

Meats and seafood were the sources for nearly 25.3% of the riboflavin in the average Polish diet (Table 7). The highest sources of riboflavin from the meats and the seafood categories were meat products (8.9%), red meat (5.5%) and poultry (5.0%) (Table 8). When considering food products from the meats and the seafood categories, the main sources of riboflavin were processed meat products (6.6%), pork (5.1%), liver and organ meat (4.4%), chicken (4.3%) and other meat products (1.5%) (Table S19).

Meats and seafood contributed 52.3% of the niacin (Table 7) in the average Polish diet. The top contributors to niacin were the following food groups: meat products (18.8%), red meat (13.9%), poultry (13.6%), other meat (3.0%) and processed seafood (2.0%) (Table 8). When considering the detailed food products, the highest ranked food sources of niacin were processed meat products (14.4%), pork (13.0%), chicken (11.9%), liver and organ meat (2.5%) and other meat products (2.3%) (Table S20).

The vitamin B6 contribution from the meats and the seafood categories was 29.4% (Table 7). The greatest main food group contributors of vitamin B6 from meat, meat products and seafood in the average Polish diet were meat products (10.5%), poultry (9.1%) and meat (6.4%) (Table 8). When considering the food products, the top three ranking food were processed meat products (8.0%), chicken (8.0%) and pork (5.9%) (Table S21).

Meats and seafood contributed 56.0% of the vitamin B12 (Table 7) in the average Polish diet. The following three food groups contributed 39.1% of the vitamin B12: other meat (17.5%), meat products (12.3%) and processed seafood (9.3%) (Table 8). The detailed data showed the highest ranked food sources of vitamin related to liver and organ meat (16.9%), processed meat products (9.2%), pork (6.5%), other fish and shellfish products (5.7%) and chicken (4.2%) (Table S22).

Meats and seafood were the sources of nearly 9% of the vitamin E in the average Polish diet (Table 7). Vitamin E was delivered mainly by meat products (Table 8). The detailed data of the food sources of vitamin E are presented in Table S23.

### 3.5. Summary

**Figure 2:**
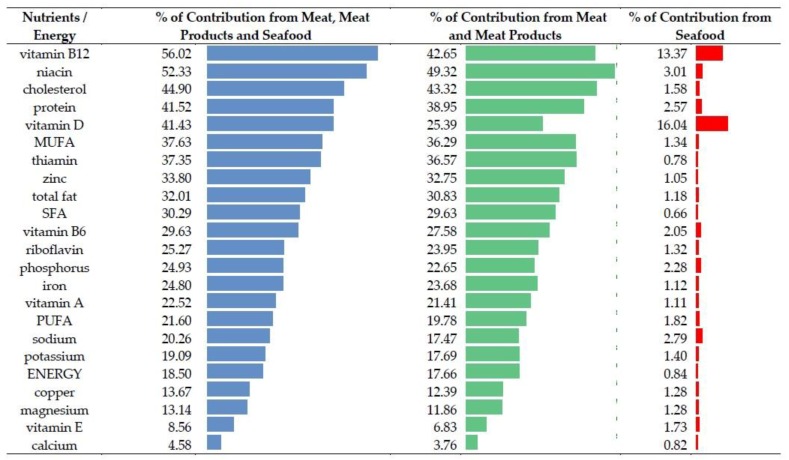
Summary: Meat, meat products and seafood as contributors of energy and nutrients in the average Polish diet. SFA—saturated fatty acids;MUFA—monounsaturated fatty acids; PUFA—polyunsaturated fatty acids.

Meat, meat products and seafood—reviewed jointly—were important sources of nutrients, delivering more than 50% of vitamin B12 and niacin (Figure 2). In the case of cholesterol, protein and vitamin D, meat, meat products and seafood were responsible for 40–50% of the daily contribution. Other nutrients (MUFA, thiamin, zinc, total fat and SFA) were delivered by 30–40% of the daily intake of meats and seafood. A lower share (20–30%) of the daily intake was observed for vitamin B6, riboflavin, phosphorus, iron, vitamin A, PUFA and sodium.

A comparison of the ranked food groups and food products from the meats and the seafood categories as the sources of energy and the 22 nutrients is presented in Table 9 and Table 10.

## 4. Discussion

Meat and meat products are an important component of Polish consumers’ diet and an important source of many nutrients. This analysis determined the contribution of meat, meat products and seafood to energy and 22 nutrients in the average Polish diet; it also ranked seven food groups and 16 food products to provide the energy and nutrient contribution. Findings of our research were compared (as listed below) with data from four studies widely discussed in the scientific literature: 2003–2006 American National Health and Nutrition Examination Survey (2003–2006 NHANES) [5], 2011–2014 American National Health and Nutrition Examination Survey (2011–2014 NHANES) [6], Australian National Nutrition and Physical Activity Survey (2011–2012 NNPAS) [37] and the study of anthropometric data, macronutrients and micronutrients intake, practice of physical activity socioeconomic data and lifestyles in Spain (2013 ANIBES) [9]. The comparison made it possible to point out the similarities and differences in energy and nutrients contribution to the diet of Polish, American, Australian and Spanish consumers. The similarities listed below indicate the homogenization of dietary patterns, whereas the differences are related with stabilized consumer preferences, price relations and the products availability. Such knowledge could be useful for health professionals to implement appropriate dietary activities and educational programmers to improve diet quality, especially among vegetarians.

Our finding indicated that the meats and the seafood categories contributed 18.5% of all the energy in the average Polish diet, while meat and meat products provided 17.6% of the energy, compared to seafood (below 1%). The detailed analyses of the energy contribution indicated some differences while compared with findings of other studies. In the average Polish diet, the highest sources of energy from meats and seafood were processed meat products (i.e., dried, boiled, salted or smoked meat of all kinds), red meat and poultry, providing 16.9% of the energy intake. In the structure of red meat consumption, the highest share was represented by pork (4.7% of the total energy intake). While the beef contribution to the energy supply amounted to 0.2% in the average Polish diet, in other population beef as an energy contributor is placed higher. For example, in the 2003–2006 NHANES, the highest ranked food groups from the meats and the seafood categories were beef (5.0% of the total energy intake), poultry (4.3%), frankfurters, sausages, luncheon meats (3.0%) and pork, ham and bacon (2.1%) [5]. According to the data from the 2011–2014 NHANES, the highest sources of energy were meat, poultry and fish in mixed dishes (4.2%); poultry (3.0%); meats (2.7%); and cured meats/poultry (2.5%) [6]. The differences in the energy sources in the diet of Polish and American consumers determined by the structure of the meat and meat products consumption are resulted from the specifics of Polish consumers, their preferences and factors determining the purchasing decision [30,32,33,58,86]. The high consumption level of processed red meat products (i.e., dried, boiled, salted or smoked pork meat), poultry (mainly chicken), pork and processed poultry products influenced the structure of the contribution of particular nutrients to the average Polish diet. In 2000–2016, the consumption of pork was 39–42 kg per capita. This level of consumption resulted from stabilized consumer preferences, favorable price relations and the availability of meat [30,58,59,87]. In contrast, the consumption of beef in 2016 amounted to 1.9 kg per person [29,31,32]. The years 2000–2012 showed a sharp drop in beef consumption from 7.1 kg to 1.2 kg, which was a consequence of price relations and the low repeatability of quality features [33,86].

Based on the comparison of our findings with the 2003–2006 NHANES and 2011–2014 NHANES, it should be pointed that the share of energy delivered by poultry and poultry products has increased. It should be treated as a positive trend because of the association of poultry consumption with the risk reduction of developing overweight and obesity, cardiovascular diseases and type 2 diabetes [25]. In Poland, the consumption structure was dominated by chicken and determined by taste, dietary aspects and favorable prices [30,32,58,87,88]. Furthermore, while comparing our findings with the 2011–2014 NHANES, it should be pointed that the share of energy delivered by seafood in the American diet (1.6%) [6] was twice as large as in the Polish diet. It is related to economic factors influencing the level of seafood consumption in Poland. The prices influenced the consumption of salmon, herring, tuna, cod, mackerel and seafood [89]. At the same time, changes in consumer behavior, increasing interest in the cuisines of other nations and increasing supply of fishes and seafood in the Polish market were observed [67,90].

When considering seven food groups and their contribution of the 22 nutrients, it should be underlined that meat products (i.e., processed red meat products, processed poultry products and other meat products) were ranked first for the contribution of 18 nutrients and energy. Meat products delivered more than 15% of the contribution for seven nutrients, namely niacin, cholesterol, protein, MUFA, thiamin, zinc, sodium and potassium. In the case of vitamin B6, iron, total fat and SFA, the contribution of meat products was 10–15% of the average daily intake, while for riboflavin, phosphorus and potassium, it was 5–10%. However, processed red meat consumption is associated with an increased risk of type 2 diabetes [4,16,17] and coronary heart disease [50] and may increase all-cause mortality [23,24]. Some findings underlined the long-term increasing consumption of red and processed meat leading to the increased risk of total mortality, cardiovascular disease, colorectal cancer and type 2 diabetes [10].

Meats were an important source of water-soluble B vitamins delivering a higher percentage of these nutrients than of energy in the average Polish diet. The contribution of meats and seafood amounted to 56.0% of vitamin B12 (meats and meat products: 42.7%, seafood: 13.3%), 52.3% of niacin (meats and meat products: 49.3%, seafood: 3.0%), 37.4% of thiamin (meats and meat products: 36.6%, seafood: 0.8%), 29.6% of vitamin B6 (meats and meat products: 27.6%, seafood: 2.0%) and 25.3% of riboflavin (meats and meat products: 24.0%, seafood: 1.3%). Based on the comparison of our studies with other findings, the high position of meat and meat products as contributors of water-soluble B vitamins has been underlined in the analysis conducted in other populations [6,8,9]; however, some differences have been observed. For example, in our study the main sources of vitamin B12 were liver and organ meat (16.9%); processed meat products, pork, other fish and shellfish products and chicken, provided nearly 43% of vitamin B12. The highest position of liver and organ meat was related to the specifics of Polish consumers and the relatively high level of consumption of organ meat and organ meat products, such as the pluck sausages [91]. In research conducted among American population, organ meat was ranked at a further position as a source of vitamin B12. In the 2003–2006 NHANES, the highest ranked food groups from the meats and the seafood categories were: (1) beef, (2) fish and shellfish, (3) frankfurters, sausages and luncheon meats, (4) organ meats, pork, ham and bacon and (5) poultry, delivering nearly 43% of vitamin B12 daily contribution [5]. According to data from the 2011–2014 NHANES, the highest sources of vitamin B12 were meats, seafood, meat, poultry and fish in mixed dishes, cured meats and poultry, delivering nearly 37% of vitamin B12 contribution [6].

With respect to niacin meats and seafood contributed 52.3% of the total intake with the main food groups: meat products (18.8%), red meat (13.9%) and poultry (13.6%). The detailed analyses pointed out the following food products: processed red meat products (14.4%), pork (13.0%) and chicken (11.9%). At the same time, findings of the niacin contribution to the American and Australian diets indicated poultry (15.4%), beef (9.2%) and pork, ham and bacon (4.3%) (2003–2006 NHANES) [5]; poultry (9.6%); meat, poultry and fish in mixed dishes (6.5%); meats (6.4%) and cured meats/poultry (4.9%) (2011–2014 NHANES) [6]; meat and meat products (34.6%) and fish (12.2%) (2013 ANIBES) [9]. The thiamin contribution from meats and seafood was 36.6% with the food groups: meat products (16.5%), red meat (16.0%) and poultry (2.7%). The highest ranked detailed food products were as follows: pork (15.8%) and processed red meat (14.7%), comparing with the other findings of the American and Australian studies: pork, ham and bacon (8.0%); and frankfurters, sausages, luncheon meats (2.9%) (2003–2006 NHANES) [5]; meat, poultry and fish in mixed dishes (4.1%); cured meat/poultry (3.6%); and poultry (3.2%) (2011–2014 NHANES) [6]; and meat and meat products (28.2%) and fish (3.5%) (2013 ANIBES) [9]. In the average Polish diet, the contribution of vitamin B6 was 29.6% from processed red meat products (8%), chicken (8%) and pork (5.9%) as compared to the other findings: poultry (9.5%), beef (8.6%), pork, ham, bacon (3.9%) and frankfurters, sausages, luncheon meats (2.8%) (2003–2006 NHANES) [5]; poultry (8.5%), meat, poultry and fish in mixed dishes (5.5%), meats (5.4%) and cured meat/poultry (3.5%) (2011–2014 NHANES) [6]; and meat and meat products (26.6%) and fish (9.1%) (2013 ANIBES) [9]. The main sources of riboflavin were processed red meat products (6.6%), pork (5.1%), liver and organ meat (4.4%) and chicken (4.3%). In other findings, the highest sources of riboflavin were beef (3.6%), poultry (3.1%), pork, ham, bacon (2.3%) and frankfurters, sausages, luncheon meats (2.1%) (2003–2006 NHANES) [5]; meats (3.8%), meat, poultry, fish in mixed dishes (3.3%), poultry (2.7%) and cured meat/poultry (2.1%) (2011–2014 NHANES) [6]; meat and meat products (32.2%) and fish (4.7%) (2013 ANIBES) [9]. These findings of Polish, American and Australian studies related to meat and meat products as sources of B vitamins should be taken into consideration in the evaluation of the quality of vegetarian diets.

Our findings indicated that meats and seafood contributed 41.5% of protein with meat products (17.4%), red meat (9.9%) and poultry (9.7%). Other findings reported the protein contribution of meats at the level of 49% in the Australian diet (2011–2012 NNPAS) [37] and identified following sources of protein: poultry (14.4%), beef (14.0%), pork, ham, bacon (5.7%), fish and shellfish (5.0%), and frankfurters, sausages, luncheon meats (4.4%) in the American diet (2003–2006 NHANES) [5].

With respect to zinc, phosphorus, iron, sodium and potassium, meats provided a higher percentage of these nutrients than the percentage of energy contribution. This indicates that meat and meat products have a higher density of these nutrients. Meats and seafood contributed 33.8% of zinc, 24.8% of iron, 20.3% of sodium and 19.1% of potassium. The four main sources of iron were meat products, red meat, poultry and other meat, delivering 23.7% of total iron intake. Other findings underline that meat and meat products are high sources of bioavailable hem [12,37,92,93,94]. Findings from the 2013 ANIBES [92] indicated that the highest sources of iron were meat, sausages and other meat product and poultry. According to the 2011–2012 NNPAS, meat/poultry/fish contributed approximately 26% of iron [37]. In the average Polish diet, meats and seafood contributed 33.8% of zinc with main food groups (meat products, red meat, poultry and other meat) delivering 32.8% of total zinc. Other findings indicated that the zinc contribution was approximately 38% from meat/poultry/fish (2011–2012 NNPAS) in the Spanish diets [37] and 35% from meats and fish, including 29% from meat and meats products and 6% from fish (2013 ANIBES) in the Australian diets [94]. The main food sources of sodium in the average Polish diet were meat products (15.2% of total contribution), including processed meat products (12.4%). Other findings indicated that processed meat was the main contributor to daily sodium intake, representing 8% of the total sodium intake per capita [95]. It should be underlined that excessive dietary sodium intake increases blood pressure and risk of hypertension, cardiovascular disease and kidney disease, as is widely discussed in the literature [95,96,97,98,99,100,101,102,103,104].

Total fat, cholesterol, MUFA, SFA and PUFA are delivered by meats and seafood in a higher percentage than the energy contribution. Meats and seafood contributed 44.9% of cholesterol, 37.6% of MUFA, 32.0% of total fat, 30.3% of SFA and 21.6% of PUFA. In the average Polish diet, the main food sources of cholesterol were meat products (16.3% of the total cholesterol intake), poultry (11.8%) and red meat (8.3%). In the case of the food products, the main sources of cholesterol were processed meat products (12.3%), chicken (10.4%) and pork (7.7%). Other findings (2011–2014 NHANES) indicated that meat contributed 42% to the total cholesterol intake (12% for poultry, 12% for mixed dishes, 8% for red meat, 5% for processed meat and 5% for seafood) [105]. For SFA, meats and seafood contributed 30.3% to the average Polish diet as compared to 29% reported in the average Australian diet according to the 2011–2012 NNPAS [37]. The three main food groups (meat products, meat and poultry) delivered 28.4% of SFA. Findings of the American studies indicated that the main food sources of SFA were beef (9.1%), frankfurters, sausages and luncheon meats (6.7%), poultry (4.2%) and pork, ham and bacon (3.5%) (2003–2006 NHANES) [5]; meat, poultry, fish in mixed dishes (4.6%), cured meats/poultry (4.6%), meats (4.0%) and poultry (2.6%) (2011–2014 NHANES) [6].

## 5. Conclusions

In conclusion, this study showed that meat and meats products are an important source of energy and nutrients. The highest share of contribution (>50%) was observed in the case of vitamin B12 and niacin. The meats and the seafood categories provided 25–50% of the average daily intake of cholesterol, protein, vitamin D, MUFA, thiamin, zinc, total fat, SFA, vitamin B6 and riboflavin. Processed red meat products (e.g., ham, sausages, salami, bacon and pepperoni) were ranked first in the contribution of thiamin, niacin, MUFA (>15% of the daily intake) and protein, total fat, SFA, cholesterol, zinc and sodium (10–15% of the daily intake). These results should be taken into consideration while analyzing the benefits and the risks of the current level of consumption of red and processed meat, fish and other seafood. Moreover, they could be useful to implement certain dietary guidelines via nutritional education and to tailor dietary recommendations to the needs of different consumer groups. The knowledge of the role of particular food groups in the energy and nutrients contribution allows to compose a sustainable diet and to monitor the nutrients that should be limited (such as SFA and sodium).

## Figures and Tables

**Table 1:** Food grouping for the analysis of energy and nutrient sources.

Food Category	Food Group	Food Product
meat and meat products	meat	(1) beef
		(2) veal
		(3) pork
		(4) sheep, goat
	poultry	(5) chicken (chicken, hen, cock)
		(6) poultry excluding chicken (turkey, duck, goose, others)
	other meat	(7) rabbit, boar, others
		(8) liver, organ meat
		(9) mixed minced meat
	meat products	(10) processed red meat products (dried, boiled, salted or smoked meat in all kinds excluding poultry e.g., ham, sausages, salami, bacon, pepperoni)
		(11) processed poultry products
		(12) other meat products
seafood	fishes	(13) fresh, chilled or frozen fish
	shellfish	(14) fresh, chilled or frozen shellfish
	processed seafood	(15) dried, smoked and salted seafood (fishes and shellfish)
		(16) other fish and shellfish products (other preserved or processed fish and shellfish preparations, canned fish and shellfish)

**Table 2:** Food group sources of energy (kcal) contribution from meat, meat products and seafood in the average Polish diet.

Food Group	Rank	% of Total Energy Contribution	Cumulative % of Total Energy Contribution
meat products	1	8.29	8.29
red meat	2	4.93	13.22
poultry	3	3.63	16.85
other meat	4	0.80	17.66
processed seafood	5	0.61	18.26
fishes	6	0.23	18.49
shellfish	7	0.01	18.50
meat, meat products and seafood		18.50	

**Table 3:** The share of meat, meat products and seafood in contribution of protein, total fat, SFA, MUFA, PUFA and cholesterol in the average Polish diet.

	Share of Meat, Meat Products and Seafood (in %)	Share of Meat and Meat Products (in %)	Share of Seafood (in %)
in contribution of:			
protein	41.52	38.95	2.57
total fat	32.01	30.83	1.18
SFA	30.29	29.63	0.66
MUFA	37.63	36.29	1.34
PUFA	21.60	19.78	1.82
cholesterol	44.90	43.32	1.58

SFA—saturated fatty acids; MUFA—monounsaturated fatty acids; PUFA—polyunsaturated fatty acids.

**Table 4:** Main food groups as the sources of protein, total fat, SFA, MUFA, PUFA and cholesterol contribution to the average Polish diet.

Nutrient	Main Food Group	% of Contribution
protein	meat products	17.44
	red meat	9.91
	poultry	9.71
total fat	meat products	14.69
	red meat	8.94
	poultry	5.87
SFA	meat products	13.99
	red meat	9.69
	poultry	4.68
MUFA	meat products	17.96
	red meat	10.18
	poultry	6.66
PUFA	meat products	7.63
	poultry	6.14
	red meat	4.87
cholesterol	meat products	16.27
	poultry	11.77
	red meat	8.29

SFA—saturated fatty acids; MUFA—monounsaturated fatty acids; PUFA—polyunsaturated fatty acids.

**Table 5:** The share of meat, meat products and seafood in the contribution of iron, zinc, sodium, phosphorus, calcium, copper and magnesium in the average Polish diet.

	Share of Meat, Meat Products and Seafood (in %)	Share of Meat and Meat Products (in %)	Share of Seafood (in %)
in contribution of:			
iron	24.80	23.68	1.12
zinc	33.80	32.75	1.05
sodium	20.26	17.47	2.79
potassium	19.09	17.69	1.40
phosphorus	24.93	22.65	2.28
calcium	4.58	3.76	0.82
copper	13.67	12.39	1.28
magnesium	13.14	11.86	1.28

**Table 6:** Main food groups as the sources of iron, zinc, sodium, phosphorus, calcium, copper and magnesium contribution in the average Polish diet.

Nutrient	Main Food Group	% of Contribution
iron	meat products	10.43
	red meat	4.66
	poultry	4.33
zinc	meat products	15.49
	red meat	9.40
	poultry	5.56
sodium	meat products	15.15
	processed seafood	2.70
	poultry	0.84
potassium	meat products	6.68
	red meat	5.17
	poultry	5.03
phosphorus	meat products	8.96
	poultry	6.89
	red meat	5.44
calcium	meat products	1.71
	red meat	0.94
	poultry	0.75
copper	meat products	4.72
	other meat	3.13
	poultry	2.32
magnesium	meat products	4.87
	poultry	3.69
	red meat	2.76

**Table 7:** The share of meat, meat products and seafood in contribution of vitamin A, vitamin D, thiamin, riboflavin, niacin, vitamin B6, vitamin B12 and vitamin E in the average Polish diet.

	Share of Meat, Meat Products and Seafood (in %)	Share of Meat and Meat Products (in %)	Share of Seafood (in %)
in contribution of:			
vitamin A	22.52	21.41	1.11
vitamin D	41.43	25.39	16.04
thiamin	37.35	36.57	0.78
riboflavin	25.27	23.95	1.32
niacin	52.33	49.32	3.01
vitamin B6	29.63	27.58	2.05
vitamin B12	56.02	42.65	13.37
vitamin E	8.56	6.83	1.73

**Table 8:** Main food groups as sources of vitamins contribution from meat, meat products and seafood in the average Polish diet.

Nutrient	Main Food Group	% of Contribution
vitamin A	other meat	17.30
	meat products	3.26
	poultry	0.82
vitamin D	processed seafood	10.96
	meat products	8.88
	poultry	8.74
thiamin	meat products	16.50
	red meat	15.98
	poultry	2.71
riboflavin	meat products	8.89
	red meat	5.47
	poultry	4.99
niacin	meat products	18.79
	red meat	13.93
	poultry	13.59
vitamin B6	meat products	10.53
	poultry	9.11
	red meat	6.42
vitamin B12	other meat	17.54
	meat products	12.27
	processed seafood	9.29
vitamin E	meat products	3.36
	poultry	1.80
	red meat	1.36

**Table 9:** Summary: Rankings of food groups as contributors of energy and nutrients ^1/^ in the average Polish diet.

Food Group	Energy ^2/^	Protein	Total fat	SFA	MUFA	PUFA	Cholesterol	Iron	Zinc	Sodium	Potassium	Phosphorus	Calcium	Copper	Magnesium	Vitamin A	Vitamin D	Thiamin	Riboflavin	Niacin	Vitamin B6	Vitamin B12	Vitamin E
red meat	2	2	2	2	2	1	3	2	2	5	2	3	2	4	3	6	4	2	2	2	3	4	2
poultry	3	3	3	3	3	2	2	6	3	3	3	2	3	3	2	3	3	3	3	3	2	5	3
other meat	4	4	4	4	4	3	4	4	4	4	4	5	5	2	5	1	6	4	4	4	4	1	4
meat products	1	1	1	1	1	6	1	5	1	1	1	1	1	1	1	2	2	1	1	1	1	2	1
fishes	6	6	6	6	6	5	6	3	6	6	6	6	6	6	6	5	5	6	6	6	6	6	6
shellfish	7	7	7	7	7	7	7	7	7	7	7	7	7	7	7	7	7	7	7	7	7	7	7
processed seafood	5	5	5	5	5	4	5	1	5	2	5	4	4	5	4	4	1	5	5	5	5	3	5

^1/^ percentage of energy and nutrients contribution from particular food groups:
  >15%  10–15%  5–10% <5%

^2/^ the number for each nutrient and energy denotes its place in the ranking according to the % of contribution.

SFA—saturated fatty acids; MUFA—monounsaturated fatty acids; PUFA—polyunsaturated fatty acids.

**Table 10:** Summary: Rankings of food products as contributors of energy and nutrients ^1/^ in the average Polish diet.

Food Product	Energy ^2/^	Protein	Total fat	SFA	MUFA	PUFA	Cholesterol	Iron	Zinc	Sodium	Potassium	Phosphorus	Calcium	Copper	Magnesium	Vitamin A	Vitamin D	Thiamin	Riboflavin	Niacin	Vitamin B6	Vitamin B12	Vitamin E
beef	11	11	12	8	11	12	11	7	7	11	10	11	11	11	11	10	10	12	10	10	11	10	11
veal	14	14	15	15	15	16	15	15	15	14	14	14	15	15	15	12	14	13	13	14	14	15	15
pork	7	2	2	2	2	3	3	3	2	8	2	3	2	3	3	16	3	1	2	2	3	3	3
sheep, goat	16	16	14	14	14	15	16	16	16	16	16	16	16	16	16	15	15	16	16	16	16	16	16
chicken	3	3	3	3	3	2	2	4	3	5	3	2	3	4	2	3	1	3	4	3	2	5	2
poultry excluding chicken	2	7	7	7	6	7	7	8	6	9	6	7	10	9	6	8	8	9	7	7	7	9	10
liver, organ meat	5	5	5	5	5	5	4	2	4	6	7	6	6	2	9	1	9	5	3	4	6	1	8
minced meat	12	12	10	10	10	11	12	12	10	12	12	12	12	13	12	13	12	6	12	12	12	13	12
other meat	13	13	13	13	13	13	13	13	14	13	13	13	13	14	13	14	13	14	14	13	13	12	14
processed meat products	1	1	1	1	1	1	1	1	1	1	1	1	1	1	1	7	2	2	1	1	1	2	1
processed poultry products	6	6	6	6	7	8	6	6	8	3	5	5	7	7	4	9	11	7	6	6	5	11	9
other meat products	4	4	4	4	4	4	5	5	5	4	4	4	5	5	5	2	7	4	5	5	4	8	5
fresh, chilled or frozen fish	10	8	11	12	12	10	9	10	11	10	8	9	9	10	8	5	6	8	11	9	8	6	7
fresh, chilled or frozen shellfish	15	15	16	16	16	14	14	14	13	15	15	15	14	12	14	11	16	15	15	15	15	14	13
dried, smoked and salted seafood	9	10	9	11	9	9	10	11	12	2	11	10	8	8	10	6	4	10	9	11	10	7	6
other fish and shellfish products	8	9	8	9	8	6	8	9	9	7	9	8	4	6	7	4	5	11	8	8	9	4	4

^1/^ percentage of energy and nutrients contribution from particular food product:
  >15%  10–15%  5–10% <5%

^2/^ the number for each nutrient and energy denoted its place in the ranking according to the % of contribution.

SFA—saturated fatty acids; MUFA—monounsaturated fatty acids; PUFA—polyunsaturated fatty acids.

## Supplementary Materials

The following Tables are available online at http://www.mdpi.com/2072-6643/10/10/1412/s1: Table S1. Food group and product sources of energy contribution from meat, meat products, seafood in the average Polish diet (food groups and products contributing <0.1% not reported); Table S2. Food group and product sources of protein contribution from meat, meat products, seafood in the average Polish diet; Table S3. Food group and product sources of total fat contribution from meat, meat products, seafood in the average Polish diet (food groups and products contributing <0.1% not reported); Table S4. Food group and product sources of SFA contribution from meat, meat products, seafood in the average Polish diet (food groups and products contributing <0.1% not reported); Table S5. Food group and product sources of MUFA contribution from meat, meat products, seafood in the average Polish diet (food group and product contributing <0.1% not reported); Table S6. Food group and product sources of PUFA contribution from meat, meat products, seafood in the average Polish diet (food group and product contributing <0.1% not reported); Table S7. Food group and product sources of cholesterol contribution from meat, meat products, seafood in the average Polish diet; Table S8. Food group and product sources of iron contribution from meat, meat products, seafood in the average Polish diet; Table S9. Food group and product sources of zinc contribution from meat, meat products, seafood in the average Polish diet; Table S10. Food group and product sources of sodium contribution from meat, meat products, seafood in the average Polish diet (food products contributing <0.1% not reported); Table S11. Food grous and product sources of potassium contribution from meat, meat products, seafood in the average Polish diet (food groups and groups contributing <0.1% not reported); Table S12. Food group and product sources of potassium contribution from meat, meat products, seafood in the average Polish diet; Table S13. Food group and product sources of calcium contribution from meat, meat products, seafood in the average Polish diet (food groups and products contributing <0.1% not reported); Table S14. Food group and product sources of copper contribution from meat, meat products, seafood in the average Polish diet (food groups and products contributing <0.1% not reported); Table S15. Food group and product sources of magnesium contribution from meat, meat products, seafood in the average Polish diet (food groups and groups contributing <0.1% not reported); Table S16. Food group and product sources of vitamin A contribution from meat, meat products, seafood in the average Polish diet (food groups and products contributing <0.1% not reported); Table S17. Food group and product sources of vitamin D contribution from meat, meat products, seafood in the average Polish diet (food groups and products contributing <0.1% not reported); Table S18. Food group and product sources of thiamin contribution from meat, meat products, seafood in the average Polish diet (food groups and products contributing <0.1% not reported); Table S19. Food group and product sources of riboflavin contribution from meat, meat products, seafood in the average Polish diet (food groups and products contributing <0.1% not reported); Table S20. Food group and product sources of niacin contribution from meat, meat products, seafood in the average Polish diet; Table S21. Food group and product sources of vitamin B6 contribution from meat, meat products, seafood in the average Polish diet (food groups and products contributing <0.1% not reported); Table S22. Food group and product sources of vitamin B12 contribution from meat, meat products, seafood in the average Polish diet; Table S23. Food group and product sources of vitamin E contribution from meat, meat products, seafood in the average Polish diet (food groups and products contributing <0.1% not reported).
