# Supplementary material for: Meat, Meat Products and Seafood as Sources of Energy and Nutrients in the Average Polish Diet
Source: Nutrients. 2018 Oct 2;10(10):1412. doi: 10.3390/nu10101412 (PMC6213018; doi:10.3390/nu10101412)
Supplement: Supplementary file 1 [file nutrients-10-01412-s001.pdf]

Meat, Meat Products and Seafood as Sources of Energy and Nutrients in the Average Polish Diet

Supplemental Tables

Table S1. Food group and product sources of energy contribution from meat, meat products, seafood in the average Polish diet (food groups and products contributing <0.1% not reported).
Table S2. Food group and product sources of protein contribution from meat, meat products, seafood in the average Polish diet.
Table S3. Food group and product sources of total fat contribution from meat, meat products, seafood in the average Polish diet (food groups and products contributing <0.1% not reported).
Table S4. Food group and product sources of SFA contribution from meat, meat products, seafood in the average Polish diet (food groups and products contributing <0.1% not reported).
Table S5. Food group and product sources of MUFA contribution from meat, meat products, seafood in the average Polish diet (food groups and products contributing <0.1% not reported).
Table S6. Food group and product sources of PUFA contribution from meat, meat products, seafood in the average Polish diet (food groups and products contributing <0.1% not reported).
Table S7. Food group and product sources of cholesterol contribution from meat, meat products, seafood in the average Polish diet.
Table S8. Food group and product sources of iron contribution from meat, meat products, seafood in the average Polish diet.
Table S9. Food group and product sources of zinc contribution from meat, meat products, seafood in the average Polish diet.
Table S10. Food group and product sources of sodium contribution from meat, meat products, seafood in the average Polish diet (food groups and products contributing <0.1% not reported).
Table S11. Food group and product sources of potassium contribution from meat, meat products, seafood in the average Polish diet (food groups and products contributing <0.1% not reported).
Table S12. Food group and product sources of potassium contribution from meat, meat products, seafood in the average Polish diet.
Table S13. Food group and product sources of calcium contribution from meat, meat products, seafood in the average Polish diet (food groups and products contributing <0.1% not reported).
Table S14. Food group and product sources of copper contribution from meat, meat products, seafood in the average Polish diet (food groups contributing <0.1% not reported).
Table S15. Food group and product sources of magnesium contribution from meat, meat products, seafood in the average Polish diet (food groups and products contributing <0.1% not reported).
Table S16. Food group and product sources of vitamin A contribution from meat, meat products, seafood in the average Polish diet (food groups and products contributing <0.1% not reported).
Table S17. Food group and product sources of vitamin D contribution from meat, meat products, seafood in the average Polish diet (food groups and products contributing <0.1% not reported).
Table S18. Food group and product sources of thiamin contribution from meat, meat products, seafood in the average Polish diet (food groups and products contributing <0.1% not reported).
Table S19. Food group and product sources of riboflavin contribution from meat, meat products, seafood in the average Polish diet (food groups and products contributing <0.1% not reported).
Table S20. Food group and product sources of niacin contribution from meat, meat products, seafood in the average Polish diet.
Table S21. Food group and product sources of vitamin B6 contribution from meat, meat products, seafood in the average Polish diet (food groups and products contributing <0.1% not reported).
Table S22. Food group and product sources of vitamin B12 contribution from meat, meat products, seafood in the average Polish diet.
Table S23. Food group and product sources of vitamin E contribution from meat, meat products, seafood in the average Polish diet (food groups and products contributing <0.1% not reported).

Table S1. Food group and product sources of energy (kcal) contribution from meat, meat products, and seafood in the average Polish diet.

Food Group<br>Food Product [1/]	Rank	% of Energy Contribution	Cumulative % of Energy Contribution
**Food Group**			
meat products	1	8.29	8.29
red meat	2	4.93	13.22
poultry	3	3.63	16.85
other meat	4	0.80	17.66
processed seafood	5	0.61	18.26
fishes	6	0.23	18.49
shellfish	7	0.01	18.50
**Food Product**			
processed red meat products	1	6.82	6.82
pork	2	4.73	11.55
chicken	3	3.20	14.76
other meat products	4	0.99	15.75
liver, organ meat	5	0.65	16.40
processed poultry products	6	0.47	16.88
poultry excluding chicken	7	0.43	17.30
other fish and shellfish products	8	0.36	17.66
dried, smoked and salted seafood	9	0.25	17.91
fresh, chilled or frozen fish	10	0.23	18.14
beef	11	0.19	18.33
minced meat	12	0.13	18.46

[1/] food groups or products contributed at least 0.1% of energy.

Table S2. Food group and product sources of protein contribution from meat, meat products, and seafood in the average Polish diet.

Food Group<br>Food Product	Rank	% of Protein Contribution	Cumulative % of Protein Contribution
**Food Group**			
meat products	1	17.44	17.44
red meat	2	9.91	27.35
poultry	3	9.71	37.06
other meat	4	1.89	38.95
processed seafood	5	1.56	40.50
fishes	6	0.99	41.49
shellfish	7	0.02	41.52
**Food Products**			
processed red meat products	1	14.00	14.00
pork	2	9.18	23.18
chicken	3	8.48	31.66
other meat products	4	1.98	33.64
liver, organ meat	5	1.51	35.15
processed poultry products	6	1.46	36.61
poultry excluding chicken	7	1.22	37.84
fresh, chilled or frozen fish	8	0.99	38.82
other fish and shellfish products	9	0.85	39.67
dried, smoked and salted seafood	10	0.71	40.38
beef	11	0.68	41.06
minced meat	12	0.30	41.36
other meat	13	0.09	41.44
veal	14	0.04	41.49
fresh, chilled or frozen shellfish	15	0.02	41.51
sheep, goat	16	0.01	41.52

Table S3. Food group and product sources of total fat contribution from meat, meat products, and seafood in the average Polish diet.

Food Group<br>Food Product [1/]	Rank	% of Total Fat Contribution	Cumulative % of Total Fat Contribution
**Food Group**			
meat products	1	14.69	14.69
red meat	2	8.94	23.63
poultry	3	5.87	29.51
other meat	4	1.32	30.83
processed seafood	5	0.95	31.78
fishes	6	0.23	32.01
**Food Product**			
processed red meat products	1	12.50	12.50
pork	2	8.70	21.20
chicken	3	5.22	26.42
other meat products	4	1.52	27.94
liver, organ meat	5	1.06	29.02
processed poultry products	6	0.67	29.69
poultry excluding chicken	7	0.65	30.34
other fish and shellfish products	8	0.57	30.91
dried, smoked and salted seafood	9	0.38	31.29
minced meat	10	0.23	31.52
fresh, chilled or frozen fish	11	0.23	31.75
beef	12	0.22	31.97
other meat	13	0.03	32.00
sheep, goat	14	0.01	32.01

[1/] food groups or products contributed at least 0.1% of total fat.

Table S4. Food group and product sources of SFA contribution from meat, meat products, and seafood in the average Polish diet.

Food Group<br>Food Product [1/]	Rank	% of SFA Contribution	Cumulative % of SFA Contribution
**Food Group**			
meat products	1	13.99	13.99
meat	2	9.69	23.68
poultry	3	4.68	28.36
other meat	4	1.27	29.63
processed seafood	5	0.52	30.15
fishes	6	0.14	30.29
**Food Product**			
processed red meat products	1	12.14	12.14
pork	2	9.38	21.52
chicken	3	4.18	25.70
other meat products	4	1.28	26.98
liver, organ meat	5	0.99	27.97
processed poultry products	6	0.57	28.54
poultry excluding chicken	7	0.50	29.04
beef	8	0.29	29.33
other fish and shellfish products	9	0.28	29.61
minced meat	10	0.26	29.86
dried, smoked and salted seafood	11	0.24	30.10
fresh, chilled or frozen fish	12	0.14	30.24
other meat	13	0.03	30.27
sheep, goat	14	0.01	30.28

[1/] food groups or products contributed at least 0.1% of SFA.

Table S5. Food group and product sources of MUFA contribution from meat, meat products, and seafood in the average Polish diet.

Food Group<br>Food Product [1/]	Rank	% of MUFA Contribution	Cumulative % of MUFA Contribution
**Food Group**			
meat products	1	17.96	17.96
red meat	2	10.18	28.15
poultry	3	6.66	34.81
other meat	4	1.48	36.29
processed seafood	5	1.11	37.40
fishes	6	0.23	37.63
**Food Product**			
processed red meat products	1	15.31	15.31
pork	2	9.92	25.23
chicken	3	5.87	31.10
other meat products	4	1.88	32.97
liver, organ meat	5	1.19	34.16
poultry excluding chicken	6	0.79	34.96
processed poultry products	7	0.77	35.73
other fish and shellfish products	8	0.65	36.38
dried, smoked and salted seafood	9	0.45	36.83
minced meat	10	0.28	37.11
beef	11	0.25	37.37
fresh, chilled or frozen fish	12	0.23	37.60
other meat	13	0.01	37.61
sheep, goat	14	0.01	37.62
veal	15	0.01	37.63

[1/] food groups or products contributed at least 0.1% of MUFA.

Table S6. Food group and product sources of PUFA contribution from meat, meat products, and seafood in the average Polish diet.

Food Group<br>Food Product [1/]	Rank	% of PUFA Contribution	Cumulative % of PUFA Contribution
**Food Group**			
meat products	1	7.63	7.63
poultry	2	6.14	13.77
red meat	3	4.87	18.63
processed seafood	4	1.45	20.09
other meat	5	1.15	21.23
fishes	6	0.36	21.59
shellfish	7	0.01	21.60
**Food Product**			
processed red meat products	1	5.80	5.80
chicken	2	5.30	11.10
pork	3	4.81	15.91
other meat products	4	1.20	17.12
liver, organ meat	5	1.00	18.11
other fish and shellfish products	6	0.98	19.09
poultry excluding chicken	7	0.84	19.93
processed poultry products	8	0.63	20.56
dried, smoked and salted seafood	9	0.47	21.03
fresh, chilled or frozen fish	10	0.36	21.38
minced meat	11	0.11	21.49
beef	12	0.05	21.54
other meat	13	0.05	21.58
fresh, chilled or frozen shellfish	14	0.01	21.59

[1/] food groups or products contributed at least 0.1% of PUFA.

Table S7. Food group and product sources of cholesterol contribution from meat, meat products, and seafood in the average Polish diet.

Food Group<br>Food Product [1/]	Rank	% of Cholesterol Contribution	Cumulative % of Cholesterol Contribution
**Food Group**			
meat products	1	16.27	16.27
poultry	2	11.77	28.04
red meat	3	8.29	36.33
other meat	4	5.99	42.32
processed seafood	5	1.64	43.95
fishes	6	0.90	44.86
shellfish	7	0.04	44.90
**Food Product**			
processed red meat products	1	12.33	12.33
chicken	2	10.41	22.73
pork	3	7.66	30.40
liver, organ meat	4	5.66	36.06
other meat products	5	2.35	38.41
processed poultry products	6	1.59	40.00
poultry excluding chicken	7	1.37	41.37
other fish and shellfish products	8	0.98	42.35
fresh, chilled or frozen fish	9	0.90	43.25
dried, smoked and salted seafood	10	0.66	43.91
beef	11	0.58	44.49
minced meat	12	0.26	44.74
other meat	13	0.07	44.81
fresh, chilled or frozen shellfish	14	0.04	44.85
veal	15	0.04	44.89
sheep, goat	16	0.01	44.90

Table S8. Food group and product sources of iron contribution from meat, meat products, and seafood in the average Polish diet.

Food Group<br>Food Product	Rank	% of Iron Contribution	Cumulative % of Iron Contribution
**Food Group**			
meat products	3	10.43	10.43
red meat	2	4.66	15.10
poultry	5	4.33	19.43
other meat	4	4.25	23.68
processed seafood	6	0.81	24.49
fishes	1	0.27	24.77
shellfish	7	0.04	24.80
**Food Product**			
processed red meat products	1	7.72	7.72
liver, organ meat	2	3.99	11.71
pork	3	3.95	15.67
chicken	4	3.77	19.43
other meat products	5	1.90	21.33
processed poultry products	6	0.82	22.15
beef	7	0.67	22.82
poultry excluding chicken	8	0.56	23.38
other fish and shellfish products	9	0.55	23.93
fresh, chilled or frozen fish	10	0.27	24.20
dried, smoked and salted seafood	11	0.26	24.46
minced meat	12	0.17	24.64
other meat	13	0.09	24.72
fresh, chilled or frozen shellfish	14	0.04	24.76
veal	15	0.03	24.79
sheep, goat	16	0.01	24.80

Table S9. Food group and product sources of zinc contribution from meat, meat products, and seafood in the average Polish diet.

Food Group<br>Food Product	Rank	% of Zinc Contribution	Cumulative % of Zinc Contribution
**Food Group**			
meat products	1	15.49	15.49
red meat	2	9.40	24.90
poultry	3	5.56	30.45
other meat	4	2.30	32.75
processed seafood	5	0.67	33.43
fishes	6	0.28	33.71
shellfish	7	0.09	33.80
**Food Product**			
processed red meat products	1	12.90	12.90
pork	2	8.48	21.38
chicken	3	4.46	25.84
liver, organ meat	4	1.92	27.76
other meat products	5	1.73	29.49
poultry excluding chicken	6	1.10	30.58
beef	7	0.88	31.46
processed poultry products	8	0.86	32.32
other fish and shellfish products	9	0.48	32.80
minced meat	10	0.30	33.10
fresh, chilled or frozen fish	11	0.28	33.39
dried, smoked and salted seafood	12	0.20	33.58
fresh, chilled or frozen shellfish	13	0.09	33.68
other meat	14	0.08	33.75
veal	15	0.04	33.79
sheep, goat	16	0.01	33.80

Table S10. Food group and product sources of sodium contribution from meat, meat products, and seafood in the average Polish diet.

Food Group<br>Food Product [1/]	Rank	% of Sodium Contribution	Cumulative % of Sodium Contribution
**Food Group**			
meat products	1	15.15	15.15
processed seafood	2	2.70	17.84
poultry	3	0.84	18.68
other meat	4	0.75	19.43
red meat	5	0.74	20.17
fishes	6	0.08	20.25
shellfish	7	0.01	20.26
**Food Product**			
processed red meat products	1	12.44	12.44
dried, smoked and salted seafood	2	1.97	14.41
processed poultry products	3	1.36	15.77
other meat products	4	1.35	17.12
chicken	5	0.73	17.85
liver, organ meat	6	0.73	18.58
other fish and shellfish products	7	0.72	19.30
pork	8	0.69	19.98
poultry excluding chicken	9	0.12	20.10
fresh, chilled or frozen fish	10	0.08	20.18
beef	11	0.04	20.22
minced meat	12	0.02	20.24
other meat	13	0.01	20.25

[1/] food groups or products contributed at least 0.1% of sodium.

Table S11. Food group and product sources of potassium contribution from meat, meat products, and seafood in the average Polish diet.

Food Group<br>Food Product [1/]	Rank	% of Potassium Contribution	Cumulative % of Potassium Contribution
**Food Group**			
meat products	1	6.68	6.68
red meat	2	5.17	11.86
poultry	3	5.03	16.88
other meat	4	0.81	17.69
processed seafood	5	0.80	18.49
fishes	6	0.58	19.08
shellfish	7	0.01	19.09
**Food Product**			
processed red meat products	1	4.88	4.88
pork	2	4.78	9.66
chicken	3	4.27	13.93
other meat products	4	0.95	14.87
processed poultry products	5	0.85	15.72
poultry excluding chicken	6	0.76	16.49
liver, organ meat	7	0.61	17.10
fresh, chilled or frozen fish	8	0.58	17.68
other fish and shellfish products	9	0.50	18.18
beef	10	0.37	18.55
dried, smoked and salted seafood	11	0.30	18.85
minced meat	12	0.15	19.00
other meat	13	0.05	19.05
veal	14	0.02	19.07
fresh, chilled or frozen shellfish	15	0.01	19.08

[1/] food groups or products contributed at least 0.1% of potassium.

Table S12. Food group and product sources of phosphorus contribution from meat, meat products, and seafood in the average Polish diet.

Food Group<br>Food Product [1/]	Rank	% of Phosphorus Contribution	Cumulative % of Phosphorus Contribution
**Food Group**			
meat products	1	8.96	8.96
poultry	2	6.89	15.86
red meat	3	5.44	21.30
processed seafood	4	1.49	22.79
other meat	5	1.35	24.14
fishes	6	0.77	24.91
shellfish	7	0.02	24.93
**Food Product**			
processed red meat products	1	6.56	6.56
chicken	2	5.91	12.46
pork	3	4.96	17.42
other meat products	4	1.24	18.66
processed poultry products	5	1.16	19.82
liver, organ meat	6	1.13	20.96
poultry excluding chicken	7	0.99	21.95
other fish and shellfish products	8	0.83	22.78
fresh, chilled or frozen fish	9	0.77	23.55
dried, smoked and salted seafood	10	0.66	24.21
beef	11	0.45	24.66
minced meat	12	0.16	24.83
other meat	13	0.05	24.88
veal	14	0.03	24.90
fresh, chilled or frozen shellfish	15	0.02	24.92
sheep, goat	16	0.01	24.93

Table S13. Food group and product sources of calcium contribution from meat, meat products, and seafood in the average Polish diet.

Food Group<br>Food Product [1/]	Rank	% of Calcium Contribution	Cumulative % of Calcium Contribution
**Food Group**			
meat products	1	1.71	1.71
red meat	2	0.94	2.64
poultry	3	0.75	3.40
processed seafood	4	0.68	4.07
other meat	5	0.36	4.44
fishes	6	0.14	4.57
shellfish	7	0.01	4.58
**Food Product**			
processed red meat products	1	1.04	1.04
pork	2	0.88	1.93
chicken	3	0.69	2.62
other fish and shellfish products	4	0.53	3.15
other meat products	5	0.44	3.59
liver, organ meat	6	0.33	3.92
processed poultry products	7	0.23	4.15
dried, smoked and salted seafood	8	0.15	4.29
fresh, chilled or frozen fish	9	0.14	4.43
poultry excluding chicken	10	0.06	4.49
beef	11	0.05	4.54
minced meat	12	0.02	4.56
other meat	13	0.01	4.57
fresh, chilled or frozen shellfish	14	0.01	4.58

[1/] food groups or products contributed at least 0.1% of calcium.

Table S14. Food group and product sources of copper contribution from meat, meat products, and seafood in the average Polish diet.

Food Group<br>Food Product [1/]	Rank	% of Copper Contribution	Cumulative % of Copper Contribution
**Food Group**			
meat products	1	4.72	4.72
other meat	2	3.13	7.85
poultry	3	2.32	10.17
red meat	4	2.22	12.39
processed seafood	5	0.89	13.28
fishes	6	0.31	13.58
shellfish	7	0.08	13.67
**Food Product**			
processed red meat products	1	3.29	3.29
liver, organ meat	2	3.02	6.31
pork	3	2.00	8.31
chicken	4	1.99	10.30
other meat products	5	0.93	11.23
other fish and shellfish products	6	0.52	11.75
processed poultry products	7	0.50	12.25
dried, smoked and salted seafood	8	0.37	12.62
poultry excluding chicken	9	0.33	12.95
fresh, chilled or frozen fish	10	0.31	13.25
beef	11	0.18	13.44
fresh, chilled or frozen shellfish	12	0.08	13.52
minced meat	13	0.07	13.59
other meat	14	0.04	13.63
veal	15	0.03	13.66

[1/] food groups or products contributed at least 0.1% of copper contribution.

Table S15. Food group and product sources of magnesium contribution from meat, meat products, and seafood in the average Polish diet.

Food Group<br>Food Product [1/]	Rank	% of Magnesium Contribution	Cumulative % of Magnesium Contribution
**Food Group**			
meat products	1	4.87	4.87
poultry	2	3.69	8.56
red meat	3	2.76	11.32
processed seafood	4	0.80	12.12
other meat	5	0.54	12.66
fishes	6	0.46	13.12
shellfish	7	0.01	13.14
**Food Product**			
processed red meat products	1	3.32	3.32
chicken	2	3.13	6.45
pork	3	2.50	8.95
processed poultry products	4	0.80	9.75
other meat products	5	0.75	10.50
poultry excluding chicken	6	0.56	11.06
other fish and shellfish products	7	0.48	11.54
fresh, chilled or frozen fish	8	0.46	12.00
liver, organ meat	9	0.43	12.43
dried, smoked and salted seafood	10	0.32	12.75
beef	11	0.24	12.99
minced meat	12	0.08	13.08
other meat	13	0.03	13.11
fresh, chilled or frozen shellfish	14	0.01	13.12
veal	15	0.01	13.13

[1/] food groups or products contributed at least 0.1% of magnesium.

Table S16. Food group and product sources of vitamin A contribution from meat, meat products, and seafood in the average Polish diet.

Food Group Food Product [1/]	Rank	% of Vitamin A Contribution	Cumulative % of Vitamin A Contribution
**Food Group**			
other meat	1	17.30	17.30
meat products	2	3.26	20.55
poultry	3	0.82	21.37
processed seafood	4	0.73	22.10
fishes	5	0.38	22.47
red meat	6	0.04	22.51
shellfish	7	0.01	22.52
**Food Product**			
liver, organ meat	1	17.29	17.29
other meat products	2	3.01	20.30
chicken	3	0.68	20.97
other fish and shellfish products	4	0.46	21.43
fresh, chilled or frozen fish	5	0.38	21.81
dried, smoked and salted seafood	6	0.27	22.07
processed red meat products	7	0.15	22.23
poultry excluding chicken	8	0.14	22.37
processed poultry products	9	0.10	22.47
beef	10	0.03	22.50
fresh, chilled or frozen shellfish	11	0.01	22.51

[1/] food groups or products contributed at least 0.1% of vitamin A.

Table S17. Food group and product sources of vitamin D contribution from meat, meat products, and seafood in the average Polish diet.

Food Group<br>Food Product [1/]	Rank	% of Vitamin D Contribution	Cumulative % of Vitamin D Contribution
**Food Group**			
processed seafood	1	10.96	10.96
meat products	2	8.88	19.85
poultry	3	8.74	28.59
red meat	4	6.82	35.41
fishes	5	5.07	40.48
other meat	6	0.95	41.43
**Food Product**			
chicken	1	7.96	7.96
processed red meat products	2	7.40	15.36
pork	3	6.48	21.84
dried, smoked and salted seafood	4	5.78	27.61
other fish and shellfish products	5	5.19	32.80
fresh, chilled or frozen fish	6	5.07	37.87
other meat products	7	1.24	39.11
poultry excluding chicken	8	0.78	39.89
liver, organ meat	9	0.72	40.61
beef	10	0.33	40.95
processed poultry products	11	0.24	41.19
minced meat	12	0.20	41.38
other meat	13	0.03	41.41
veal	14	0.01	41.42
sheep, goat	15	0.01	41.43

[1/] food groups or products contributed at least 0.1% of vitamin D.

Table S18. Food group and product sources of thiamin contribution from meat, meat products, and seafood in the average Polish diet.

Food Group<br>Food Product	Rank	% of Thiamin Contribution	Cumulative % of Thiamin Contribution
**Food Group**			
meat products	1	16.50	16.50
red meat	2	15.98	32.48
poultry	3	2.71	35.19
other meat	4	1.38	36.57
processed seafood	5	0.42	36.99
fishes	6	0.35	37.34
shellfish	7	0.01	37.35
**Food Product**			
pork	1	15.78	15.78
processed red meat products	2	14.73	30.51
chicken	3	2.44	32.95
other meat products	4	1.37	34.31
liver, organ meat	5	0.92	35.24
minced meat	6	0.44	35.67
processed poultry products	7	0.40	36.08
fresh, chilled or frozen fish	8	0.35	36.43
poultry excluding chicken	9	0.27	36.70
dried, smoked and salted seafood	10	0.23	36.93
other fish and shellfish products	11	0.20	37.12
beef	12	0.18	37.30
veal	13	0.02	37.32
other meat	14	0.02	37.34
fresh, chilled or frozen shellfish	15	0.01	37.35

[1/] food groups or products contributed at least 0.1% of thiamin.

Table S19. Food group and product sources of riboflavin contribution from meat, meat products, and seafood in the average Polish diet.

Food Group<br>Food Product	Rank	% of Riboflavin Contribution	Cumulative % of Riboflavin Contribution
**Food Group**			
meat products	1	8.89	8.89
red meat	2	5.47	14.36
poultry	3	4.99	19.35
other meat	4	4.63	23.98
processed seafood	5	0.93	24.90
fishes	6	0.35	25.25
shellfish	7	0.01	25.27
**Food Product**			
processed red meat products	1	6.59	6.59
pork	2	5.06	11.66
liver, organ meat	3	4.44	16.10
chicken	4	4.33	20.44
other meat products	5	1.47	21.91
processed poultry products	6	0.82	22.73
poultry excluding chicken	7	0.65	23.39
other fish and shellfish products	8	0.50	23.88
dried, smoked and salted seafood	9	0.43	24.31
beef	10	0.37	24.68
fresh, chilled or frozen fish	11	0.35	25.03
minced meat	12	0.16	25.19
veal	13	0.03	25.22
other meat	14	0.03	25.25
fresh, chilled or frozen shellfish	15	0.01	25.26

[1/] food groups or products contributed at least 0.1% of riboflavin.

Table S20. Food group and product sources of niacin contribution from meat, meat products, and seafood in the average Polish diet.

Food Group<br>Food Product	Rank	% of Niacin Contribution	Cumulative % of Niacin Contribution
**Food Group**			
meat products	1	18.79	18.79
red meat	2	13.93	32.72
poultry	3	13.59	46.31
other meat	4	3.01	49.32
processed seafood	5	1.97	51.29
fishes	6	1.02	52.31
shellfish	7	0.02	52.33
**Food Product**			
processed red meat products	1	14.41	14.41
pork	2	12.99	27.40
chicken	3	11.91	39.31
liver, organ meat	4	2.48	41.79
other meat products	5	2.28	44.06
processed poultry products	6	2.10	46.16
poultry excluding chicken	7	1.69	47.85
other fish and shellfish products	8	1.16	49.01
fresh, chilled or frozen fish	9	1.02	50.03
beef	10	0.87	50.89
dried, smoked and salted seafood	11	0.82	51.71
minced meat	12	0.41	52.12
other meat	13	0.12	52.24
veal	14	0.06	52.30
fresh, chilled or frozen shellfish	15	0.02	52.32
sheep, goat	16	0.01	52.33

Table S21. Food group and product sources of vitamin B6 contribution from meat, meat products, and seafood in the average Polish diet.

Food Group<br>Food Product	Rank	% of Vitamin B6 Contribution	Cumulative % of Vitamin B6 Contribution
**Food Group**			
meat products	1	10.53	10.53
poultry	2	9.11	19.65
red meat	3	6.42	26.07
other meat	4	1.52	27.58
processed seafood	5	1.17	28.75
fishes	6	0.87	29.62
shellfish	7	0.01	29.63
**Food Product**			
processed red meat products	1	8.03	8.03
chicken	2	8.02	16.05
pork	3	5.88	21.93
other meat products	4	1.27	23.20
processed poultry products	5	1.23	24.44
liver, organ meat	6	1.23	25.67
poultry excluding chicken	7	1.09	26.76
fresh, chilled or frozen fish	8	0.87	27.63
other fish and shellfish products	9	0.61	28.24
dried, smoked and salted seafood	10	0.56	28.80
beef	11	0.51	29.31
minced meat	12	0.19	2950
other meat	13	0.09	29.,59
veal	14	0.03	29.62
fresh, chilled or frozen shellfish	15	0.01	29.63

1/ food groups or products contributed at least 0.1% of vitamin B6.

Table S22. Food group and product sources of vitamin B12 contribution from meat, meat products, and seafood in the average Polish diet.

Food Group<br>Food Product	Rank	% of Vitamin B12 Contribution	Cumulative % of Vitamin B12 Contribution
**Food Group**			
other meat	1	17.54	17.54
meat products	2	12.27	29.81
processed seafood	3	9.29	39.10
red meat	4	7.43	46.53
poultry	5	5.41	51.94
fishes	6	3.89	55.83
shellfish	7	0.19	56.02
**Food Product**			
liver, organ meat	1	16.87	16.87
processed red meat products	2	9.20	26.07
pork	3	6.48	32.55
other fish and shellfish products	4	5.65	38.20
chicken	5	4.22	42.43
fresh, chilled or frozen fish	6	3.89	46.32
dried, smoked and salted seafood	7	3.64	49.95
other meat products	8	2.24	52.20
poultry excluding chicken	9	1.19	53.39
beef	10	0.89	54.27
processed poultry products	11	0.83	55.10
other meat	12	0.40	55.50
minced meat	13	0.27	55.78
fresh, chilled or frozen shellfish	14	0.19	55.96
veal	15	0.05	56.01
sheep, goat	16	0.01	56.02

Table S23. Food group and product sources of vitamin E contribution from meat, meat products, and seafood in the average Polish diet.

Food Group<br>Food Product	Rank	% of Vitamin E Contribution	Cumulative % of Vitamin E Contribution
**Food Group**			
meat products	1	3.36	3.36
poultry	2	1.80	5.16
red meat	3	1.36	6.51
processed seafood	4	1.32	7.83
fishes	5	0.40	8.23
other meat	6	0.32	8.55
shellfish	7	0.01	8.56
**Food Product**			
processed red meat products	1	2.35	2.35
chicken	2	1.69	4.04
pork	3	1.28	5.32
other fish and shellfish products	4	0.93	6.24
other meat products	5	0.87	7.11
dried, smoked and salted seafood	6	0.40	7.51
fresh, chilled or frozen fish	7	0.40	7.90
liver, organ meat	8	0.28	8.19
processed poultry products	9	0.14	8.33
poultry excluding chicken	10	0.10	8.43
beef	11	0.07	8.50
minced meat	12	0.03	8.54
fresh, chilled or frozen shellfish	13	0.01	8.55
other meat	14	0.01	8.56

[1/] food groups or products contributed at least 0.1% of vitamin E.
